# Exposure of Human Lung Cells to Tobacco Smoke Condensate Inhibits the Nucleotide Excision Repair Pathway

**DOI:** 10.1371/journal.pone.0158858

**Published:** 2016-07-08

**Authors:** Nathaniel Holcomb, Mamta Goswami, Sung Gu Han, Samuel Clark, David K. Orren, C. Gary Gairola, Isabel Mellon

**Affiliations:** 1 Department of Toxicology and Cancer Biology, The Markey Cancer Center, University of Kentucky, Lexington, Kentucky, United States of America; 2 Toxicology Laboratory, Department of Food Science and Biotechnology of Animal Resources, College of Animal Bioscience and Technology, Konkuk University, Seoul, Republic of Korea; King Faisal Specialist Hospital & Research center, SAUDI ARABIA

## Abstract

Exposure to tobacco smoke is the number one risk factor for lung cancer. Although the DNA damaging properties of tobacco smoke have been well documented, relatively few studies have examined its effect on DNA repair pathways. This is especially true for the nucleotide excision repair (NER) pathway which recognizes and removes many structurally diverse DNA lesions, including those introduced by chemical carcinogens present in tobacco smoke. The aim of the present study was to investigate the effect of tobacco smoke on NER in human lung cells. We studied the effect of cigarette smoke condensate (CSC), a surrogate for tobacco smoke, on the NER pathway in two different human lung cell lines; IMR-90 lung fibroblasts and BEAS-2B bronchial epithelial cells. To measure NER, we employed a slot-blot assay to quantify the introduction and removal of UV light-induced 6–4 photoproducts and cyclobutane pyrimidine dimers. We find a dose-dependent inhibition of 6–4 photoproduct repair in both cell lines treated with CSC. Additionally, the impact of CSC on the abundance of various NER proteins and their respective RNAs was investigated. The abundance of XPC protein, which is required for functional NER, is significantly reduced by treatment with CSC while the abundance of XPA protein, also required for NER, is unaffected. Both XPC and XPA RNA levels are modestly reduced by CSC treatment. Finally, treatment of cells with MG-132 abrogates the reduction in the abundance of XPC protein produced by treatment with CSC, suggesting that CSC enhances proteasome-dependent turnover of the protein that is mediated by ubiquitination. Together, these findings indicate that tobacco smoke can inhibit the same DNA repair pathway that is also essential for the removal of some of the carcinogenic DNA damage introduced by smoke itself, increasing the DNA damage burden of cells exposed to tobacco smoke.

## Introduction

Lung cancer is a deadly disease and a leading cause of cancer-related mortality in the US and in the world [1–3]. In 2012, the most recent year data is available, lung cancer accounted for 1.8 million cases of cancer and 1.6 million deaths worldwide [4, 5]. Exposure to tobacco smoke is the predominant risk factor for the development of lung cancer and it is estimated to account for 85–90% of all lung cancer cases [6, 7]. It is also associated with the formation of tumors at additional sites in the body that are not directly exposed to smoke including the bladder, pancreas, liver, stomach and bone marrow [8, 9]. Tobacco use remains prevalent in certain regions of the world [10] and while its use has declined in the US, approximately 50% of newly diagnosed lung cancers occur in former smokers [6]. Hence, lung cancer and other forms of cancer associated with tobacco smoke exposure remain a tremendous health burden in the US and world-wide. Continued elucidation of the molecular mechanisms that lead to the formation of cancers associated with tobacco smoke is essential for prevention, treatment and identification of individuals who are at greatest risk for the development of cancer.

Thousands of compounds have been identified in the vapor and particulate phases of cigarette smoke and they include carcinogens, co-carcinogens, mutagens and tumor promoters. Approximately 70 of these compounds have been classified as carcinogens [7, 11]. Different classes of chemical carcinogens are present in tobacco smoke including the polycyclic aromatic hydrocarbons (PAHs) such as benzo[a]pyrene (B[a]P), dibenz[a,h]anthracene and dibenzo[a,l]pyrene. The DNA-reactive metabolites of PAHs are considered to be among the primary tobacco smoke carcinogens [7, 12]. Metabolic activation of these and other chemical compounds found in tobacco smoke can generate intermediates that react with DNA bases and produce DNA adducts. Hence, DNA adducts are likely continually formed in the lung tissues of people who smoke, and if they are not removed by DNA repair processes, their persistence could lead to the formation of mutations. Many different types of genetic alterations are found in lung cancer and they include point mutations, genomic rearrangements, amplifications and large scale insertions and deletions. Mutations in KRAS and TP53 are frequently found in lung tumors and lung tissues of smokers [6, 13, 14], and the accumulation of mutations in these and other important oncogenes and tumor suppressor genes are driving forces in the development of lung cancer.

PAH-induced DNA damage is removed by the nucleotide excision repair (NER) pathway [15–21] and hence, NER activity is likely critical to the prevention of carcinogen-induced mutations that contribute to neoplasia associated with smoke exposure. NER is a versatile pathway that removes a wide variety of structurally diverse DNA lesions including those generated by metabolites of chemical carcinogens as well as those generated by exposure to ultraviolet (UV) light. The cylobutane pyrimidine dimer (CPD) and 6–4 photoproduct (6–4 PP), produced by UV light, are model substrates commonly studied when measuring NER activity as they are rapidly generated by a brief exposure to UV light [22]. In mammals, at least 20 different protein factors participate in NER, including the XPA-G factors that are singly defective in the 7 corresponding complementation groups of the human disease, xeroderma pigmentosum (XP). The tumor suppressor factor p53 also impacts NER efficiency probably by transcriptional regulation of the *XPC* and *DDB2* gene products [23–26]. The NER pathway is comprised of two sub-pathways that differ in their mechanism of damage recognition: global genomic NER (GG-NER) which can remove damage from anywhere in the genome and transcription-coupled NER (TC-NER) which selectively removes damage from the transcribed strands of expressed genes. In GG-NER, DNA damage recognition is accomplished by XPC, which is stabilized by its binding partners RAD23B, and CENTRIN2 [27]. In TC-NER, damage is recognized by the stalling of the RNA polymerase complex at the site of damage (reviewed in [28]). After DNA damage recognition, many of the subsequent steps are the same for GG-NER and TC-NER. The helicase activities of TFIIH produce additional unwinding of DNA where upon the endonuclease activities of the XPF/ERCC1 complex and XPG produce single-strand incisions flanking the damaged site. The original integrity of the DNA is restored after an approximately 30 nucleotide region of DNA containing the lesion is removed, and the gap is filled by pol δ or pol ε, using the undamaged strand as a template (reviewed in [22]).

Several different types of animal models have been used to investigate the molecular mechanisms of lung cancer development caused by exposure to tobacco smoke. Unfortunately, many smoke inhalation studies have had limited success [29–31]. The A/J mouse model has been used extensively but it is confounded by the spontaneous lung tumors in control animals, need for long smoke exposure and recovery regimens, low tumor induction by smoke inhalation and responses that do not adequately mimic those found in humans exposed to tobacco smoke [29, 32]. Greater tumor incidence has been achieved by exposing female B6C3F1 mice to lifetime, whole-body mainstream tobacco smoke [31, 33]. The utilization of mice with targeted disruptions in tumor suppressor genes or oncogenes associated with lung cancer development in humans may yield improved animal models and additional mechanistic insights [34].

As animal models for tobacco smoke exposure have had limited success, cigarette smoke condensate (CSC) has been used as a surrogate for cigarette smoke exposure to study its effects in model cell culture systems [8]. It is the particulate phase of cigarette smoke collected on Cambridge filters and resuspended in DMSO. It is mutagenic and genotoxic and produces several different types of mutations including point mutations, deletions, loss of heterozygosity (LOH), microsatellite instability, sister chromatid exchanges and micronuclei [35–40]. It introduces DNA damage [40–43]. It also induces human and mammalian cell transformation [44–46] and tumor formation when applied to mouse skin [47–49]. While it is well established that cigarette smoke introduces a variety of different types of DNA damage, it is less clear how smoke exposure influences DNA repair efficiency and DNA damage response pathways.

Loss of DNA repair capability results in increased mutagenesis and carcinogenesis. In mouse models, deficiencies in NER have been associated with tumorigenesis at many organ sites including the lung [50]. Compared to normal mice, NER-deficient mice have a higher incidence of lung tumors when exposed to B[a]P [51, 52] and XPC-deficient mice have elevated levels of spontaneous lung tumors [53]. By extension, even a partial loss of NER efficiency in people is likely to increase mutagenesis and cancer incidence, particularly in cases of chronic DNA damage induction, as occurs in the lung tissue of smokers.

We have studied the effects of CSC on the NER pathway using an immuno-slot blot assay to measure NER in two human lung cell lines; IMR-90 and BEAS-2B. We find a dose-dependent inhibition of the efficiency of NER when both cell lines are treated with increasing concentrations of CSC. Our results provide evidence for the first time that CSC can directly interfere with the normal NER process, both in terms of overall efficiency as well as at the protein and RNA level of NER factors, suggesting a possible new manner by which tobacco smoke may promote carcinogenesis.

## Materials and Methods

### Cell Culture

The human primary lung fibroblast cell line IMR-90 (obtained from the American Type Culture Collection) was grown in minimal essential medium (Eagle) containing Earle’s salts (Mediatech) supplemented with 0.1 mM non-essential amino acids (Lonza), 2 mM glutamine (Mediatech), 100 units/ml penicillin, 100 μg/ml streptomycin and 10% fetal bovine serum (Sigma). The human bronchial epithelial cell line, BEAS-2B (American Type Culture Collection), was grown in Dulbecco’s Modified Eagle’s Medium supplemented with 2 mM glutamine, 100 units/mL penicillin, 100 μg/mL streptomycin and 10% heat inactivated fetal bovine serum. Both cell lines were maintained in a 5% CO_2_ incubator at 37°C.

### Preparation of CSC

CSC was prepared using the University of Kentucky Reference 3R4F Cigarettes as previously described [38]. Briefly, the particulate phase of smoke was collected on Cambridge filters using a 30-port smoking machine (Borgwaldt) under standard Federal Trade Commission conditions, [54] dissolved in DMSO at a concentration of 40 μg/ul, and stored in small aliquots at -80°C. At the time of use, aliquots were thawed in a 37°C water bath and discarded after use.

### Treatment of Cells with CSC

IMR-90 cells were grown to confluency prior to treatment and then treated with CSC for 24 h unless otherwise stated. BEAS-2B cells were seeded at a density of 1.2 million cells per 10 cm plate, allowed to grow for 24 h and log phase cells were then treated with CSC for 16 h. The growth of BEAS-2B cells does not exhibit contact inhibition so studies could not be performed using confluent cultures. For both cell lines, control (mock treated) cells were treated with DMSO, the solvent used for the preparation of CSC, at a volume equal to that used for the highest dose of CSC in each experiment.

### Treatment with MG-132

MG-132, a potent inhibitor of proteasome mediated proteolysis, was prepared from a 2mM stock solution from EMD Bioscience (catalog # 475791) stored at -20°C. The solution was thawed immediately prior to treatment and diluted for use. To investigate how changes in the abundance of XPC protein are influenced by UV-C irradiation, confluent IMR-90 cells were treated with MG-132 for 4 h prior to irradiation. The cells were irradiated with 20 J/m^2^ UV-C and either lysed immediately or returned to medium containing MG-132 for incubation after UV. XPC levels were examined using the Western method described above. To investigate a potential involvement of the proteasome in the turnover of XPC after cells are treated with CSC, confluent IMR-90 cells were treated with 120 μg/ml CSC for 16 h in the presence or absence of MG-132. After treatment, cells were counted and lysed, and XPC levels were measured by Western Blotting.

### Analysis of Cell Viability

IMR-90 and BEAS-2B cells were grown and treated with CSC as described above. Cells were trypsinized, washed with chilled PBS, centrifuged, and resuspended in 10 mL of chilled PBS. Cells were counted in triplicate for each dose using trypan blue dye exclusion. For both cell lines, the results presented are an average of four independent biological experiments unless otherwise noted.

### Measurement of Nucleotide Excision Repair

The removal of 6-4PPs and CPDs from total genomic DNA was measured using an immunoblot assay as previously described [55, 56] with some modifications. Cells were grown and treated with CSC as described above. They were then washed twice with PBS and irradiated with UV-C light to introduce photolesions; 20 J/m^2^ UV-C to measure the removal of 6–4 PPs and 2 J/m^2^ UV-C to measure the removal of CPDs. After irradiation, cells were either lysed immediately or after incubation in medium containing DMSO or CSC for increasing periods of time to permit repair. DNA was isolated using a Promega wizard genomic DNA isolation kit. DNA concentration was measured using a fluorometer and H-dye. The DNA was then denatured and equal amounts from each sample were loaded onto a Hybond nitrocellulose membrane (Biorad) using a slot blot apparatus (100 ng of DNA per slot for the detection of 6–4 PPs and 20 ng of DNA per slot for detection of CPDs). The membrane was baked in a vacuum oven at 80°C for 1 h, treated with 5% nonfat dry milk (Blotto, Santa Cruz Biotechnology) in TBST (10 mM Tris pH 8.0, 150 mM NaCl and 0.1% Tween-20) for 1 h, and incubated overnight at 4° with mouse monoclonal antibodies (1:10,000 dilution) specific for either 6–4 PPs or CPDs (CAC-NM-DND-002, CAC-NM-DND-001, Cosmo Bio USA) in 1% dry milk and TBST. The following day, the membrane was washed extensively with TBST and then incubated with goat anti-mouse horseradish peroxide-conjugated secondary antibodies (1:10,000 dilution, Thermo Scientific) in 1% dry milk and TBST for 2 h at room temperature. After washing, chemiluminescence (ECL–Plus, GE Healthcare Bio-Sciences Corp) and fluorimaging were used to detect the photolesions. Image Quant computer software was employed to quantify the signal detected by the phosphorimager. The percent repair at each time point was calculated by dividing the signal strength of the slot-blot band obtained at each repair time point by the signal strength of the band obtained at the zero time point. For most membranes, a DNA ladder of irradiated DNA was used as an internal control for uniformity of slotting, antibody incubation and development with chemiluminescence. Unirradiated samples of DNA were also loaded to detect any nonspecific binding of the antibodies to DNA which in general was found to be insignificant.

### Western Analysis

The effect of CSC treatment on the abundance of XPC, XPA, and β-actin proteins in IMR-90 cells and BEAS-2B cells was determined by Western blotting using mouse monoclonal antibodies (XPA: sc-56813, XPC: sc-74411, Santa Cruz Biotechnology; β-actin: A3854, Sigma). IMR-90 and BEAS-2B cells were treated with different concentrations of CSC or mock treated as described above. After treatment, cells were washed with PBS and trypsinized. Approximately five million cells were collected by centrifugation for each treatment, washed with PBS and stored at -20° or -80°C. Each sample of frozen cells was thawed on ice, resuspended in 200 μl RIPA buffer (50 mM Tris (7.4), 150 mM NaCl, 1% NP-40, 1 mM EDTA, 0.25% Na-Deoxycholate) containing 0.6 mM PMSF, 1% Protease Cocktail Inhibitor (Sigma) and 2 units of DNase 1 (New England BioLabs), lysed by sonication, centrifuged at 13,500 G to remove debris, and the amount of protein in each of the supernatants was quantified using the Bradford method. Alternatively, cells were counted, pelleted, directly lysed in loading dye and samples were loaded based on cell number. Samples containing 100 μg of protein were mixed with loading dye, boiled at 100°C for 5 min, resolved in 8% (XPC/Actin) or 12% (XPA/Actin) SDS-PAGE gels and transferred to a PVDF membrane (Bio-Rad). The membranes were incubated for one h with 5% nonfat dry milk in TBST for blocking. For XPC and actin, the upper half of the membrane was probed with a 1:1,000 dilution of XPC antibody and the lower half with 1:100,000 dilution of ß Actin antibody and incubated overnight at 4° C. The membrane was then washed extensively with TBST and incubated for 2 h with 1:5,000 diluted HRP-conjugated goat anti-mouse antibodies (GE Healthcare Bio-Sciences Corp) at room temperature. After extensive washing with TBST, the binding of antibodies was detected using enhanced chemiluminescence (ECL–Plus, GE Healthcare Bio-Sciences Corp) and fluorimaging. For XPA and actin, the full membrane was probed with 1:1,000 dilution of XPA antibody overnight at 4° C. Washing and development for XPA was performed in the same manner as XPC. The membrane was then stripped using a stripping buffer (Pierce) at 37° C for 15 min, washed with TBST, blocked and probed with actin antibody. The amount of XPC or XPA in each lane was normalized to the amount of actin in the same lane.

### Real-Time PCR

The effect of CSC on the expression of XPC, XPA, and GAPDH RNA in IMR-90 cells was measured using Real-Time PCR. Confluent cells were incubated with CSC or DMSO as a control (as described), washed with PBS, trypsinized, centrifuged, washed with PBS and pelleted. Total RNA was isolated from one million cells for each treatment using a GenElute mammalian total RNA Miniprep kit (Sigma). The RNA 6000 Nano Chip kit from Agilent was used to determine the quality and concentration of RNA isolated from the cells. For each treatment, 1.5 μg of RNA was reverse transcribed to cDNA using random primers and a high capacity cDNA RT kit (Applied Biosystems). Primer sets specific to each gene were identified using Universal Probe Library (Roche, http://www.universalprobelibrary.com) and synthesized by Integrated DNA Technologies. Sequences of the primers are given in S1 Table. Reactions were carried out on a Roche LightCycler 480 system using a 96-well plate format. Each reaction (20 μl) contained 1X Master Mix, 100 nM fluorescent reporter probe, 200 nM of each forward and reverse primer and 5 μl of cDNA (diluted 1:10). Samples were first incubated for 10 min at 95°C followed by 40 cycles of amplification (95° C for 15 s denaturation and 60° C for 1 min annealing). An equal volume of 1:10 diluted cDNA from each treatment was mixed together to create the DNA used as standards. Serial dilutions of mixed cDNA (1:1, 1:10, 1:100, 1:1,000, and 1:10,000) were used to create a standard curve. The expression levels of RNA in the treated cells were determined by comparing their crossing point (CP) values with the CP values of standard curves for the corresponding gene or RNA. The crossing point is the point at which the fluorescence of a sample achieves a threshold fluorescence. The amounts of XPC or XPA RNA were normalized to the amounts of GAPDH RNA for each treatment.

### Statistical Considerations

All statistical evaluations were done using Graph Pad Prism 6. For the experiments comparing multiple treatments to a control, a 1-way ANOVA with a Holm-Sidak test for multiple-comparison was employed. For the experiments comparing one treatment to a control, a Student’s T-test was employed. For all analyses, p<0.05 was used as the threshold for significance. Statistical significance is described in the results section and not presented on the images as some graphs contained too many significant treatments to indicate on the graphs.

## Results

### CSC inhibits NER in IMR-90 cells

**Fig 1:**
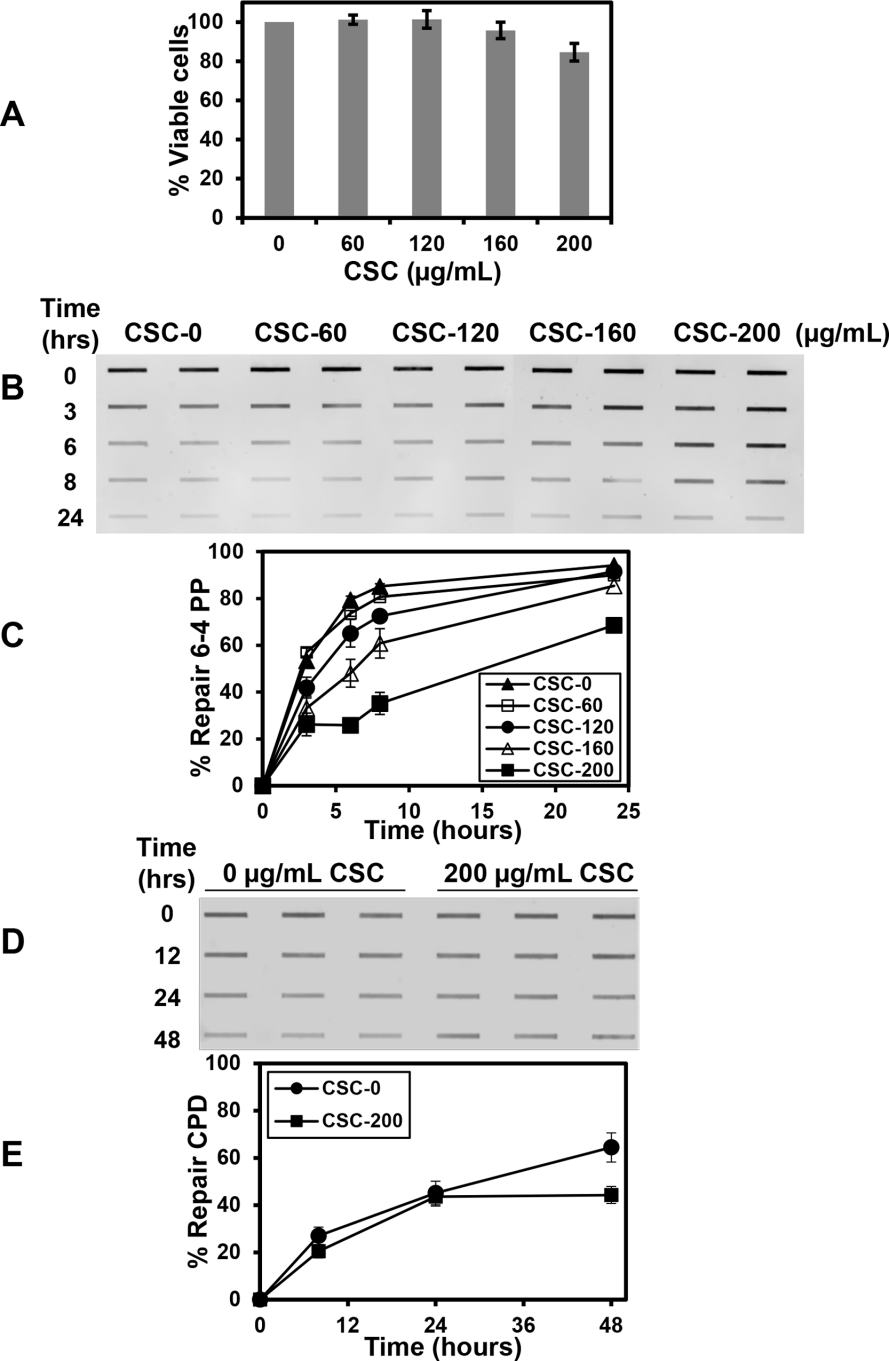
CSC inhibits NER in IMR-90 human lung fibroblasts. (A) Cell viability after CSC treatment. Confluent IMR-90 cells were treated with CSC (or mock treated with DMSO) with the concentrations shown for 24 h and the percentage of viable cells was measured using Trypan blue dye exclusion. The data represent the mean ± SE (Standard Error) from four independent experiments (except for 60 μg/ml CSC which included two independent experiments). (B) Removal of 6–4 PPs. Cells were treated with the concentrations of CSC shown or with DMSO for 24 h and irradiated with 20 J/m^2^ UVC to introduce photolesions. After irradiation, cells were either lysed immediately or after incubation in medium containing CSC or DMSO for the times (h) shown to permit repair. The immunoblot assay to detect 6–4 PPs was performed and samples were loaded in duplicate for each repair time point. (C) Removal of 6–4 PPs. A graphical representation of results obtained from multiple immunoblots measuring the removal of 6–4 PPs is shown. Each data point represents the mean ± SE of three repeats from two independent experiments. (D) Removal of CPDs. IMR-90 cells were treated with 200 μg/mL CSC or with DMSO for 24 h and irradiated with 2 J/m^2^ UVC to introduce photolesions. After irradiation, cells were either lysed immediately or after incubation in medium containing CSC or DMSO for the times shown to permit repair. An immunoblot assay to detect CPD lesions was performed and samples were loaded in triplicate for each time point. (E) Removal of CPDs. A graphical representation of results obtained from multiple immunoblots measuring the removal of CPDs is shown. Each data point represents the mean ± SE of three repeats from two independent experiments.

We first investigated the effects of CSC on the NER pathway by studying a human lung fibroblast cell line, IMR-90. The effect of CSC on cell viability was evaluated by treating cells with a range of different doses, 0 to 200 μg/ml, for 24 h. No significant effect on viability was observed even after treatment of cells with 200 μg/ml, the highest concentration of CSC used (Fig 1A). NER was studied using an immuno-slot blot assay that measures the removal of 6-4PPs or CPDs introduced by UV irradiation. These lesions are repaired exclusively by NER in human cells and 6-4PPs are model substrates to measure NER efficiency in total genomic DNA. In untreated cells, 6-4PPs are rapidly removed from DNA with the majority of lesions removed within 3 h after UV irradiation. Treatment with CSC inhibits the removal of 6-4PP lesions in a dose-dependent fashion and the inhibition was statistically significant for all doses of CSC used (except 60 μg/ml) at one or more time points (Fig 1B and 1C). In general, NER in CSC treated samples was slowed but reached completion within 24 h after UV irradiation except when cells were treated with the highest dose of CSC, where both the kinetics and extent of repair were reduced. In contrast to the rapid removal of 6-4PPs from genomic DNA found in untreated IMR-90 cells, the removal of CPDs in untreated cells was much slower. This observation of slow and inefficient removal of CPDs is similar to what other investigators have previously found in human cell lines using comparable doses of UV light [57, 58]. Treatment with CSC had only a minor inhibitory effect on the removal of CPDs (Fig 1D and 1E), with a statistically significant difference only observed at the 48 h time point (p = .0435). It is unlikely that this difference is biologically significant and interpretation of the values representing the amount of repair at the 48 h time point could be confounded by replicative DNA synthesis that occurred during the repair period. While the IMR-90 cells were studied in a confluent state, small differences in the amount of DNA replication at the 48 h time point could contribute to the differences in repair measured in the CSC-untreated and treated samples.

### CSC reduces the abundance of XPC but not XPA protein in IMR-90 cells

**Fig 2:**
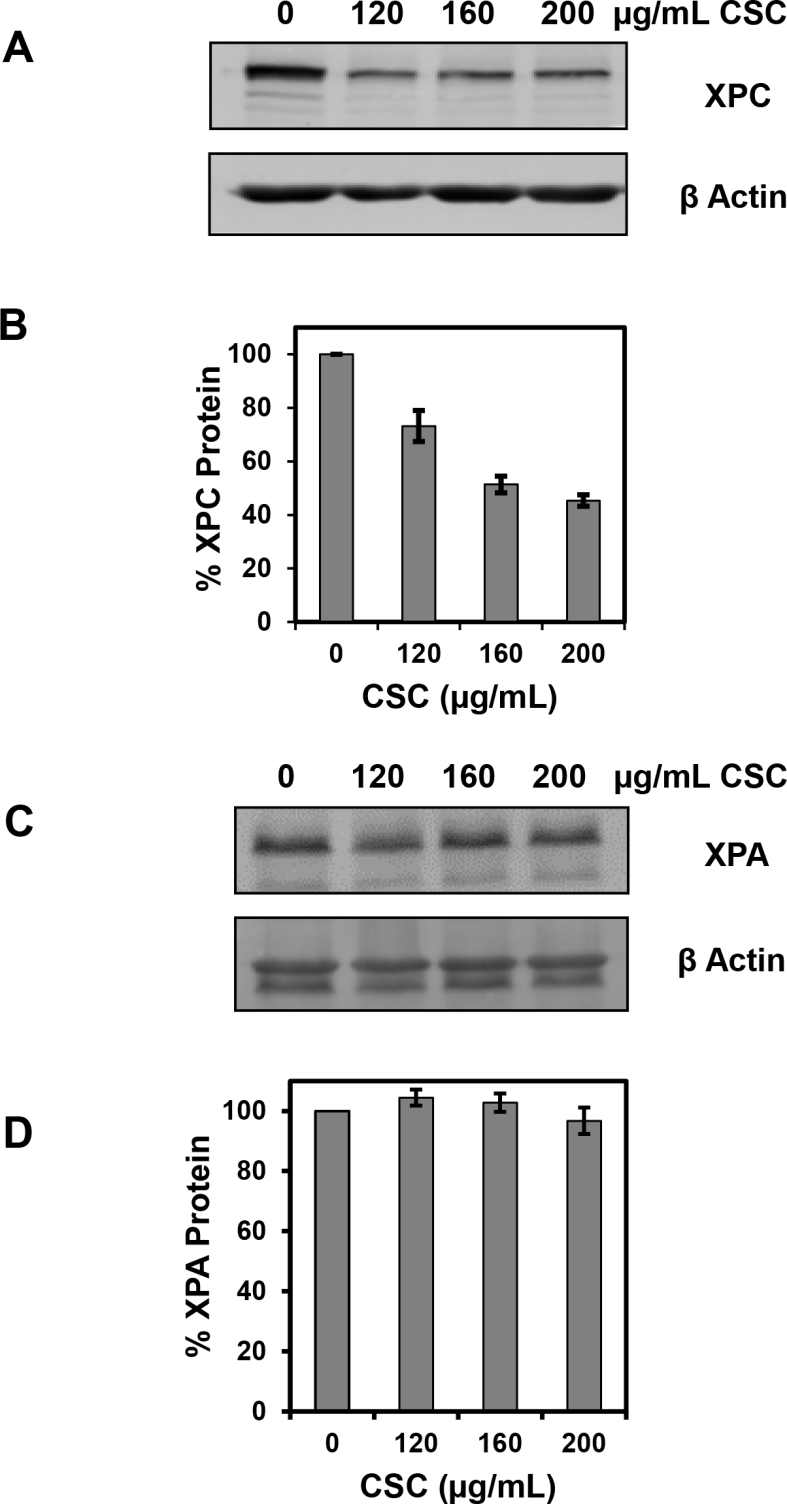
CSC reduces the abundance of XPC, but not XPA, protein in IMR-90 cells. (A) Cells were treated with different concentrations of CSC as shown for 24 h, and the abundance of XPC was examined by western blot analysis. (B) A graphical representation of multiple western blots for XPC expression is shown. The data presented are the mean ± SE from two repeats each of three independent experiments, and XPC expression was normalized to β-actin. (C) Cells were treated as shown for 24 h, and the abundance of XPA was measured by western blot analysis. (D) A graphical representation of multiple western blots for XPA expression is shown. The data presented are the mean ± SE of three repeats from three independent experiments, and XPA expression was normalized to β-actin.

The effect of CSC on the abundance of XPC and XPA protein was determined by western blotting using whole cell lysates of IMR-90 cells treated with 120, 160 and 200 μg/ml of CSC for 24 h. These are the same doses and treatment time used to study the effects of CSC on NER. Whole cell lysates from mock treated cells (DMSO) were used as controls. The abundance of XPC protein was reduced in IMR-90 cells treated with increasing concentration of CSC for 24 h (Fig 2A and 2B); XPC protein levels were reduced by 56% in cells treated with 200 μg/ml of CSC. The reduction in XPC protein compared to the mock treated cells was statistically significant in all treatments. In contrast, there was not any statistically significant change in the abundance of XPA protein in cells treated with CSC (Fig 2C and 2D). The amounts of XPC and XPA proteins were normalized to the amounts of β actin present in each lane.

### A timecourse of CSC treatment shows a correlation between the reduction of XPC protein and the inhibition of NER

**Fig 3:**
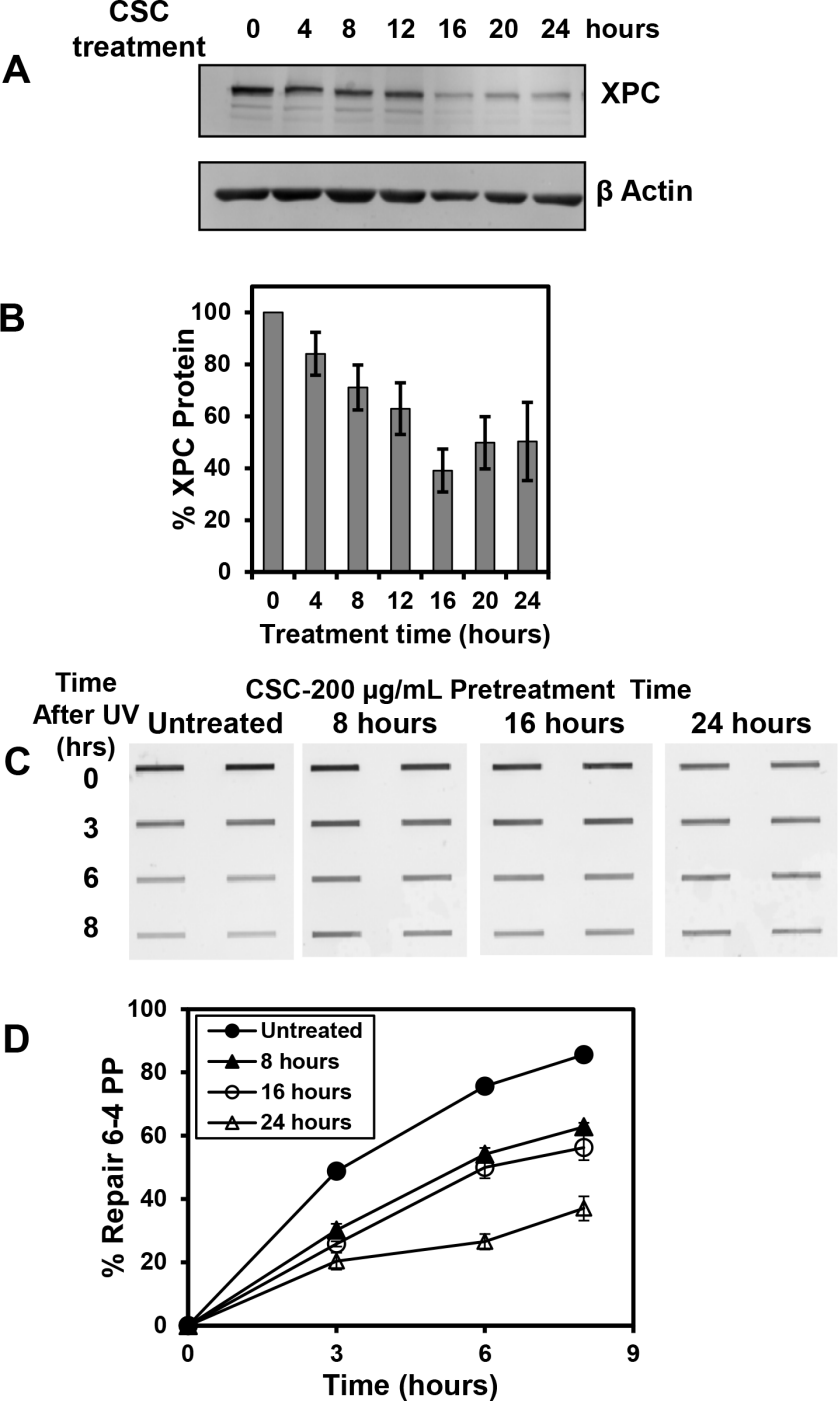
The impact of CSC on XPC protein and NER efficiency depends on treatment duration. (A) IMR-90 Cells were treated with 200 μg/ml of CSC for the times indicated and XPC expression was examined by western blot analysis. (B) A graphical representation of multiple western blots examining the time course of inhibition for XPC expression after CSC treatment is shown. The data presented are the mean ± SE of three repeats from one experiment, and XPC expression was normalized to β-actin. (C) Results of an immunoblot showing the time course of the effect of CSC on repair of 6–4 PPs in IMR-90 cells. Cells were treated with 200 μg/mL CSC for 8, 16, or 24 h (or DMSO for 24 h) and irradiated with 20 J/m^2^ UVC to introduce photolesions. After irradiation, cells were either lysed immediately or after incubation in medium containing CSC or DMSO for the times shown (3, 6 and 8 h) to permit repair. An immunoblot assay was performed and samples were loaded in duplicate to measure the removal of 6–4 PPs. (D) A graphical representation of multiple immunoblots for 6–4 repair is shown. Each data point represents the mean ± SE of three repeats from one experiment.

Treatment of IMR-90 cells with CSC for 24 h inhibits NER and results in reduced expression of XPC protein. To measure the kinetics of these inhibitions, cells were treated with 200 μg/ml of CSC and the abundance of XPC protein was measured at 4 h intervals over a 24 h period and NER was measured after treatment with CSC for 8 h intervals over a 24 h period. A reduction in XPC protein compared to untreated cells was observed as early as 8 h and it was maximally inhibited by 16 h. The inhibition was statistically significant for all timepoints except 4 h (Fig 3A and 3B). Significant inhibition of NER was observed at all treatment times beginning with the 8 h treatment (Fig 3C and 3D). NER was maximally inhibited with the longest length of treatment, 24 h, and a statistically significant difference from cells not treated with CSC was observed for all repair timepoints.

### CSC modestly reduces XPC and XPA RNA levels in IMR-90

**Fig 4:**
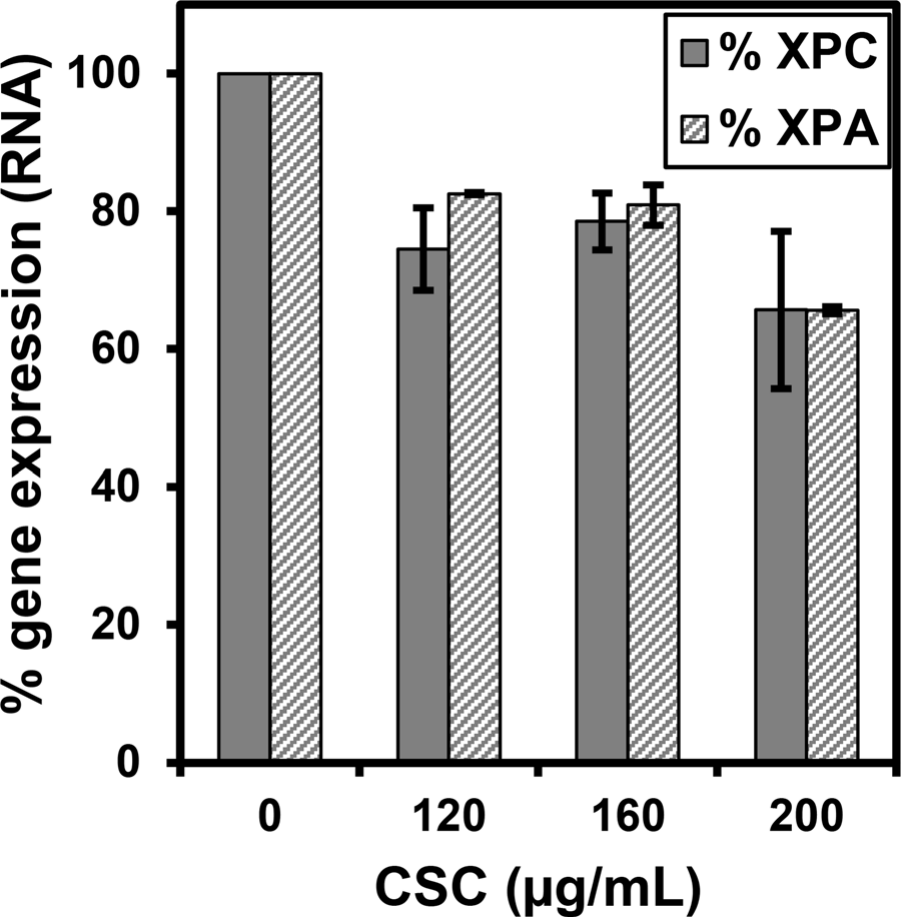
The effect of CSC on the abundance of XPC and XPA RNA in IMR-90 cells. Cells were treated with the indicated concentrations of CSC (or DMSO) for 24 hours. RNA was isolated and Real Time PCR was performed as described in the methods section. The expression of XPC or XPA RNA was normalized to GAPDH RNA for each treatment. The data presented are the mean ± SE of one analysis from three independent experiments.

The effect of CSC treatment on the abundance of XPC or XPA RNA in IMR-90 cells was measured using the same doses of CSC that were used to evaluate its effect on protein levels. No statistically significant changes in the abundance of XPC RNA were observed when cells were treated with 120 μg/ml or 160 μg/ml CSC, whereas a modest but statistically significant decrease was observed when cells were treated with 200 μg/ml of CSC (Fig 4). XPA showed modest, but significant, reduction in RNA expression across all CSC treatments.

### CSC inhibits NER and reduces the abundance of XPC protein in BEAS-2B cells

**Fig 5:**
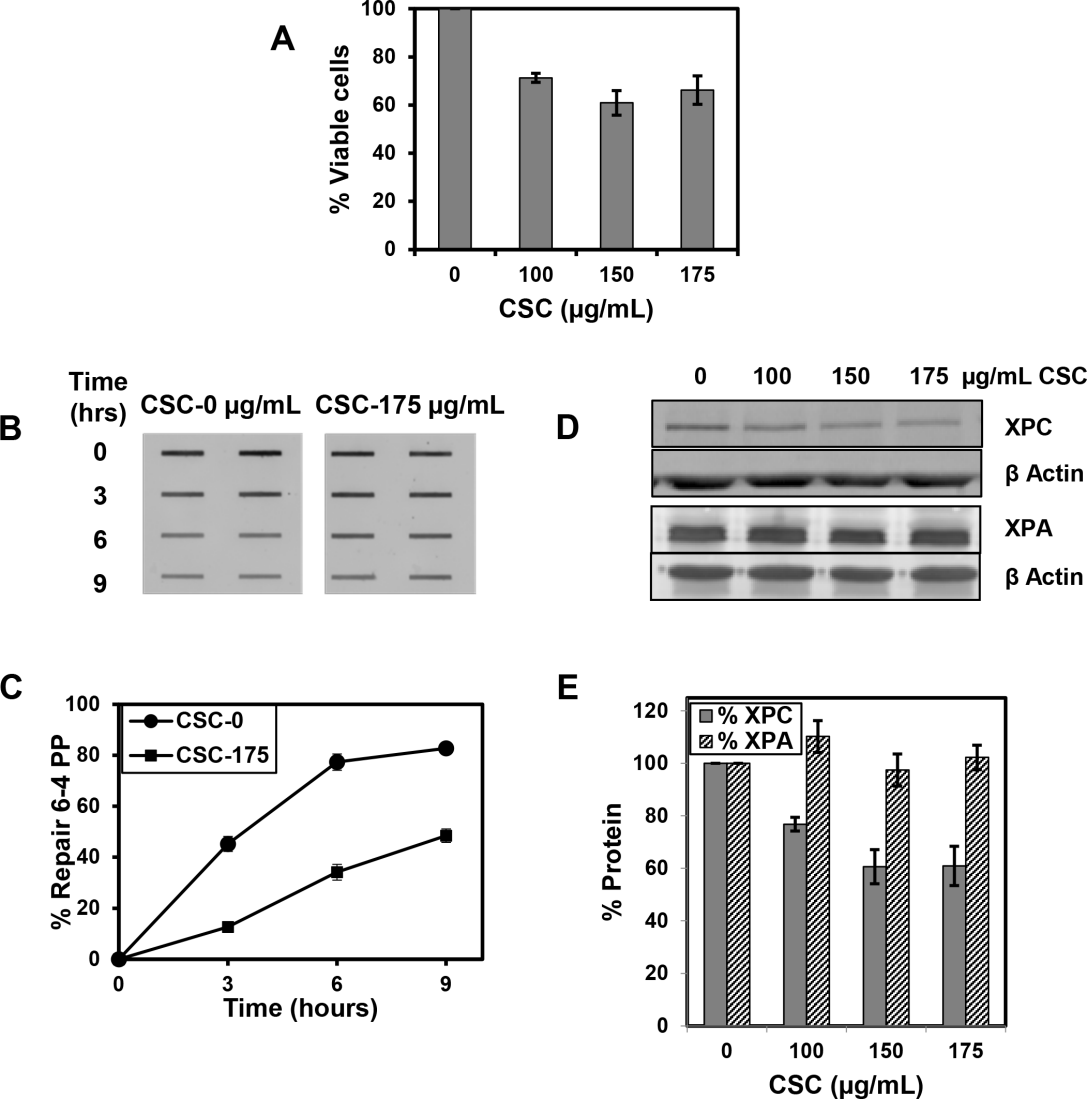
CSC inhibits NER and the abundance of XPC protein in BEAS-2B cells. (A) Cells were treated with the concentrations of CSC shown for 16 h and the percentage of viable cells was measured using Trypan blue dye exclusion. The data presented are the mean ± SE of four biological experiments. (B) Cells were treated with 175 μg/mL CSC (or DMSO) for 16 h, and irradiated with 20 J/m^2^ UVC to introduce photolesions. After irradiation, cells were either lysed immediately or after incubation in medium containing CSC or DMSO for increasing periods of time to permit repair. An immunoblot assay was performed and samples were loaded in duplicate to measure the removal of 6–4 PPs. (C) A graphical representation of multiple immunoblots for 6–4 repair is shown. Each data point represents the mean ± SE of four repeats from one experiment. (D) Cells were treated with the different concentrations of CSC shown for 24 h, lysed, and the abundance of XPC and XPA were examined by western analysis. (E) A graphical representation of multiple western blots for XPC and XPA protein is shown. The data presented are the mean ± SE of two repeats from two independent experiments for XPC and three repeats of one experiment for XPA. XPC and XPA values were normalized to β-actin.

We also investigated the impact of treatment with CSC on NER in a bronchial epithelial cell line, BEAS-2B. We chose BEAS-2B cells due to the relevance of epithelial cells as sites of lung cancer formation, as they are the cells that line the respiratory tract and directly interact with inhaled carcinogens. The effect of CSC on cell viability was evaluated by treating cells with the same dose range chosen for IMR-90 viability (Fig 5A). Some toxicity was observed for BEAS-2B cells treated with CSC compared to little or no toxicity observed in IMR-90 cells (Fig 1A). BEAS-2B were actively replicating at the time of treatment, and as a result they were likely more sensitive than IMR-90 cells to the toxicity of CSC. The effect of CSC on NER was then studied by measuring the removal of 6-4PPs after treatment with 175 ug/ml CSC for 16 h (Fig 5B). In cells not treated with CSC, NER was rapid and efficient. Treatment with CSC resulted in significant inhibition of the removal of 6–4 PPs at all three time points (Fig 5C). BEAS-2B cells were also treated with different doses of CSC for 16 h and probed for XPC and XPA protein expression (Fig 5D). A reduction in the abundance of XPC protein, but not XPA, was observed across the range of treatments of BEAS-2B cells with CSC (Fig 5E); similar to our findings in IMR-90 cells.

### The reduction of XPC protein in IMR-90 cells by CSC treatment is mediated through the proteasome

**Fig 6:**
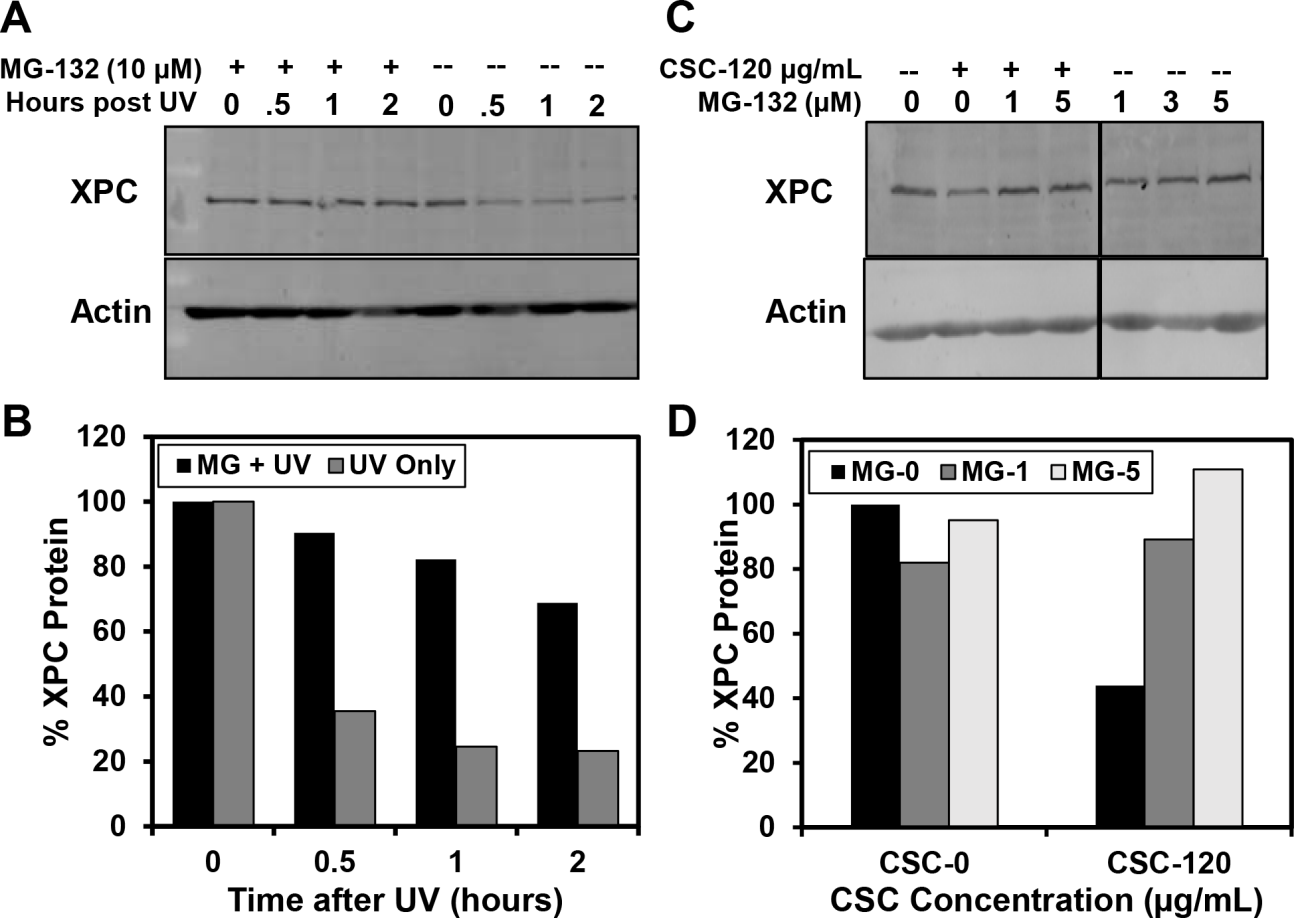
Involvement of the proteasome in the reduced expression of XPC protein in IMR-90 cells after treatment with UV or CSC. (A) Cells were treated with 10 μM MG-132 or untreated for 4 hours, and then irradiated with 20 J/m^2^ UV-C. After irradiation, cells were either lysed immediately or after incubation for increasing periods of time in the same type of medium as was used for the pretreatment; medium containing MG-132 or not containing MG-132. XPC expression was measured using Western blot analysis. (B) A graphical representation of the experiments from (A) is shown. XPC expression was normalized to β-actin. The data presented are the mean of two repeats from one experiment. The percent XPC protein was calculated by comparing post-UV time points to the appropriate 0 h (MG-treated or not MG-treated) time point. Treatment with MG-132 for 4 h had a negligible impact on XPC expression before irradiation, so both 0 h values were set to 100%. (C) Cells were treated with a combination of CSC and/or MG-132 at the indicated concentrations for 16 h. XPC levels were measured by Western blot analysis and normalized to Actin. (D) A graphical representation of the average obtained from two different blots for the experiments in (C) is shown; treatment with 3 μM MG-132 was not included. XPC expression was normalized to β-actin.

After observing that treatment of IMR-90 cells with CSC results in a significant reduction in the abundance of XPC protein, we investigated the potential influence of CSC treatment on ubiquitin mediated turnover of XPC. UV irradiation induces ubiquitination of XPC protein [59] and XPC protein abundance has been previously linked to ubiquitin modification. After UV irradiation, XPC protein levels quickly drop in a proteasome dependent manner [60–62], although this appears to be both temporary and UV-dose dependent [59]. We also addressed this by treating IMR-90 cells with MG-132, which permits ubiquitin-linkages but prevents ubiquitin-mediated proteasomal degradation, and then exposing those cells to UV irradiation. XPC levels were reduced after UV exposure, but in the presence of MG-132, this reduction was significantly diminished (Fig 6A). This confirms that UV-mediated XPC protein levels are altered in a proteasome-dependent manner. We then asked whether the observed inhibition of XPC by CSC was also mediated by the proteasome. We treated IMR-90 cells with CSC in the presence of MG-132 for 16 h and observed that the reduction in XPC protein level produced by treatment with CSC alone was almost completely abrogated by the addition of MG-132 (Fig 6B). 120 ug/ml CSC reduced XPC expression in the absence of MG-132, but in the presence of 1 and 5 μM MG-132, CSC did not inhibit XPC protein expression, suggesting that the inhibition of XPC protein expression by CSC requires functional proteasomal activity.

## Discussion

In this study, we find that cigarette smoke condensate, a surrogate for tobacco smoke exposure, can inhibit NER function and reduce the expression of XPC, a key protein required for DNA damage recognition in the NER pathway. Consequently tobacco smoke exposure can affect the integrity of DNA in two fundamentally different ways. It is well established that it can introduce DNA damage, an important contributor to lung carcinogenesis. However, our findings indicate that, in addition, it can also inhibit the DNA repair pathway that is essential for the removal of some of the carcinogenic DNA damage introduced by smoke carcinogens. Hence, cells of the lung exposed to smoke would likely suffer an even greater DNA damage burden than previously held. Certain individual constituents of tobacco smoke have been implicated in inhibiting DNA repair. Acrolein, a combustion product of cigarette smoke, has been shown to inhibit the nucleotide excision repair pathway and it inhibits XPC expression in a proteasome-dependent fashion [63–65]. In addition, arsenic, a metal constituent of tobacco smoke, has been found to reduce XPC expression [66]. Together, these studies suggest that components of tobacco smoke can impact lung carcinogenesis by inhibiting NER. CSC can also inhibit the base excision repair (BER) pathway, which repairs oxidative DNA damage, another type of tobacco smoke damage [43, 44]. Hence, it will be important to investigate how tobacco smoke-induced alterations in different DNA repair pathways contribute to lung carcinogenesis.

DNA damage recognition is an early, key step in NER and several studies suggest that DNA damage recognition by XPC is or can be the rate-limiting step in the pathway. Reduced expression of XPC has been associated with reduced repair of UV-induced photoproducts [67] and increased cancer incidence [68, 69]. Biochemical and cellular studies indicate that the binding affinity of XPC for the DNA lesion or the time it takes XPC to find the DNA lesion may be the specific rate limiting step [70–72]. Conversely, complementation of an XPA deficient cell line with very low levels of XPA protein fully restores DNA repair activity [73] and XPA becomes rate limiting only when levels are reduced by over 90% [74, 75]; these studies suggest that the participation of XPA protein is usually not a rate limiting step in NER. We find that treatment of cells with CSC inhibits NER and there is a concomitant reduction in XPC protein while XPA protein levels remain unchanged. If XPC protein is rate limiting in NER, then the inhibition of NER by CSC may be a direct consequence of the reduction in XPC protein. Additional genetic studies that manipulate the abundance of XPC during or after CSC treatment are needed to more directly establish the relationship between CSC induced alterations in XPC protein levels and its inhibition of NER. We find that treatment with CSC results in only a modest reduction in the levels of XPC RNA and it is only detected well after the amounts of XPC protein are reduced. This suggests that most of the reduction in XPC protein produced by treatment with CSC is not caused by a reduction in XPC RNA. Our observation of a reduction in XPA RNA but not XPA protein after treatment with some concentrations of CSC might be due to a relatively high abundance of XPA protein or low turnover of the protein. Lastly, an additional protein, UV DNA Damage Binding Protein 2 (UV-DDB2), is specifically required for the recognition and removal of CPDs in cells and functions in the turnover of XPC that can impact the removal of both CPDs and 6-4PPs. We attempted to examine the effect of CSC treatment on the abundance of DDB2 but were unsuccessful in identifying an antibody that yielded results specific to the protein.

Previous studies have demonstrated that XPC protein is polyubiquitinated after exposure to UV light and its polyubiquitination is mediated by the UV-DDB-Ubiquitin ligase complex [59, 61, 76]. There are at least two distinct types of ubiquitin modifications to XPC following UV-induced DNA damage. A lysine-48-linked polyubiquitin linkage appears to promote degradation of XPC by the proteasome [77]. In contrast, a lysine-63-linked ubiquitination of XPC can be critical for the removal of XPC from the lesion site and allow downstream NER factors access to the DNA damage [78]. In addition, although the type of ubiquitin linkage was not determined, ubiquitin modification of XPC can actually increase its binding affinity for undamaged DNA [59] XPC is also modified by sumoylation in response to UV damage [61] and inhibition of this modification reduces XPC stability after UV irradiation. XPC SUMO modification has been implicated in promoting lysine-63 mediated XPC polyubiquitination [78, 79]. A recent report revealed seven unique UV-induced ubiquitination sites on XPC [80]. All of these studies demonstrate the complexity of the post-translational modifications to XPC in response to DNA damage and they likely represent [81] the intricacies of regulating such an important and complex pathway.

We observe that treatment with CSC results in a reduction in the abundance of XPC protein in the absence of UV damage, and that this reduction is reversed when cells are treated with MG-132. This suggests that the reduction in the abundance of XPC protein produced by exposure to CSC is a consequence of enhanced proteasome-dependent turnover of the protein that is mediated by ubiquitination. XPC is intrinsically unstable as a monomer [82] and knocking down one of its binding partners, HR23B, promotes XPC degradation in a proteasome dependent manner [83]. Studies have also found that ubiquitination and proteasome-dependent turnover of XPC is important in maintaining steady-state levels of the protein in the absence of DNA damage [84]. In addition, the deubiquitinating enzyme USP-7 removes a UV-induced polyubiquitin chain from XPC that would otherwise target it for proteasome-mediated degradation [62]. Hence, HR23B and USP-7 both function to stabilize XPC by inhibiting ubiquitin-mediated degradation. Since treatment with CSC can result in the introduction of different forms of DNA damage (discussed below), it is possible that the induced turnover of XPC by the proteasome is mediated through the introduction of certain types of DNA damage. Additional studies are needed to determine if CSC-mediated turnover of XPC functions by promoting ubiquitination or inhibiting deubiquitination and to characterize the sites of ubiquitination. CSC has been previously shown to enhance the proteasome-mediated turnover of Akt, a protein kinase [85], which together with our studies may indicate that smoke exposure can impact multiple pathways by targeting specific proteins to the proteasome for degradation.

The assay used to measure NER in this study measures the introduction and removal of UV light-induced photoproducts. We chose to use this approach because it provides a method to rapidly introduce DNA damage and it precisely quantitates the kinetics of removal of a model substrate for the NER pathway. Moreover, the use of UV light as a DNA damaging agent is not confounded by variations in cellular uptake or metabolic activation as can be the case when using chemical agents to introduce DNA damage. It is likely that CSC would inhibit the removal of other substrates for NER but additional studies are required to investigate its effect on the removal of chemical modifications to DNA introduced by tobacco smoke. Tobacco smoke contains a number of compounds capable of producing DNA lesions, several of which are repaired by the NER pathway, including BPDE. The BPDE lesion can be particularly important in the etiology of smoking-related lung cancer, as the G-T transversion signature mutation of BPDE [8, 81, 86–88] is found more frequently in at CpG mutational hotspots in the *P53* gene of lung tumors from smokers compared with lung tumors from non-smokers [89–91]. Mice exposed topically to B[a]P develop skin tumors with the same signature mutations in p53 [92]. Hence, measuring the effect of CSC on the removal of BPDE adducts is important to directly demonstrate that tobacco smoke exposure inhibits the repair of DNA damage introduced by the tobacco smoke itself. In our study, it is likely that any adducted base damage directly introduced by the CSC treatment was very small compared to the levels of photoproducts introduced by UV irradiation. For example, a previous study found that treatment of rat buccal mucosal cells for 24 h with doses of CSC similar to those used in our study, introduced approximately 2 DNA modifications per 1 x 10^8^ bases [93]. Only a subset of this small number would likely serve as substrates for NER. In contrast, the dose of UV light used in our studies introduces approximately 5,000 times more adducts than what has been estimated for CSC induced DNA damage and all of these UV-induced lesions are potential substrates for NER [94]. Thus, it is unlikely that the inhibition of NER by CSC treatment was due to a competition between different types of DNA damage introduced during the experiments or titration of the NER pathway by damage directly introduced by CSC.

## Conclusions

Cigarette smoking remains the highest risk factor for lung cancer development in the United States and the world. However, not all smokers develop lung cancer. Our results suggest that CSC, a commonly accepted surrogate for tobacco smoke exposure, inhibits the NER pathway, increasing the persistence of lesions in DNA. In addition, CSC reduces the abundance of XPC protein, a key protein required for DNA damage recognition in NER, and this reduction is likely produced by targeting XPC to the proteasome for degradation. It is well established that variations in DNA repair capacity contribute to cancer risk, and our findings indicate that inhibition of NER is another mechanism by which smoking may contribute to development of lung cancer. Therefore, it may be prudent to consider measuring individual DNA repair capacity as a means of evaluating lung cancer risk, particularly among people who have other risk factors.

## Supporting Information

S1 Table Primers used in quantitative real-time PCR analysis. Multiple primer pairs were used to measure XPC and XPA RNA expression, and the resulting analysis did not produce any measurable difference between primer pairs within a single gene, so both primer pairs were included in the analysis of these genes.
(TIF)
