# Supplementary figures and images for: Exposure of Human Lung Cells to Tobacco Smoke Condensate Inhibits the Nucleotide Excision Repair Pathway
Source: PLoS One. 2016 Jul 8;11(7):e0158858. doi: 10.1371/journal.pone.0158858 (PMC4938567; doi:10.1371/journal.pone.0158858)

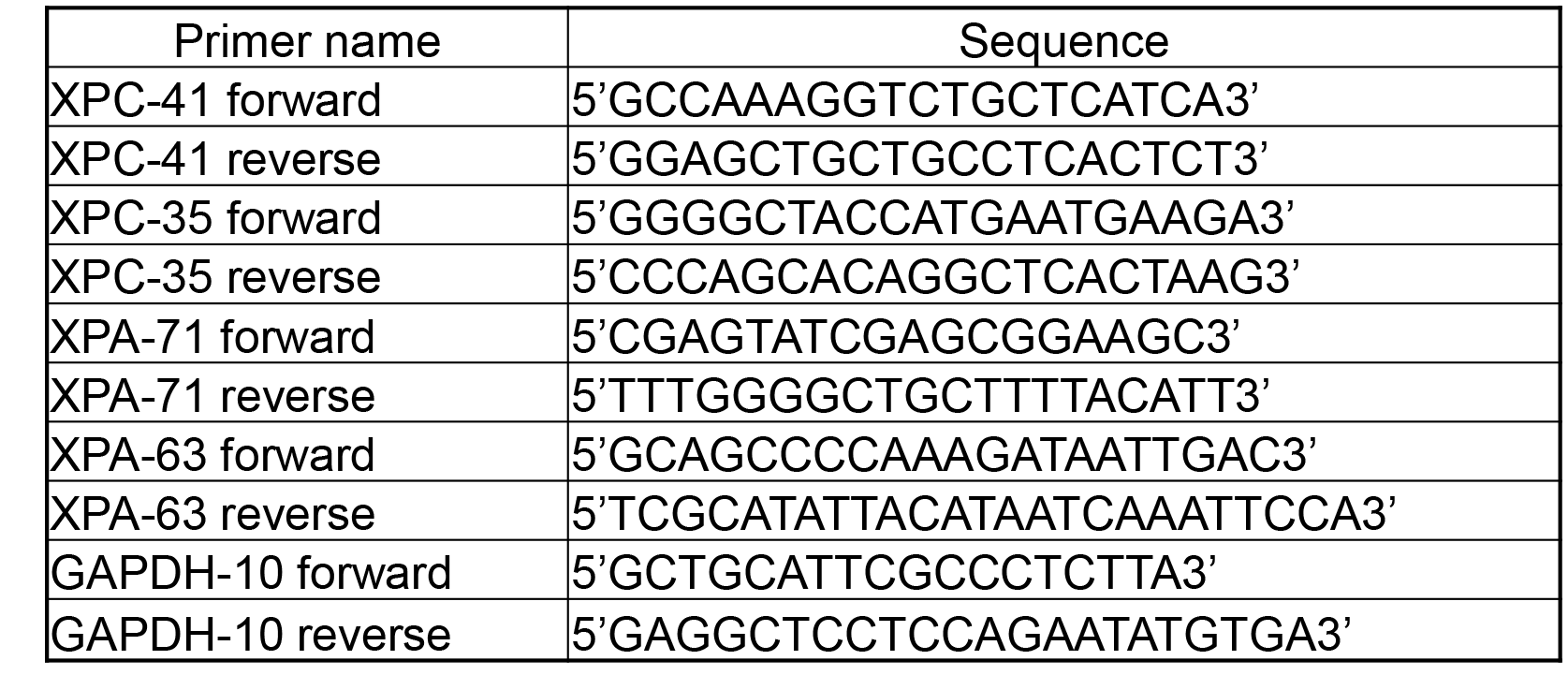

Supplement: S1 Table — Multiple primer pairs were used to measure XPC and XPA RNA expression, and the resulting analysis did not produce any measurable difference between primer pairs within a single gene, so both primer pairs were included in the analysis of these genes. (TIF) [file pone.0158858.s001.tif]
